# Paroxysmal ventricular tachycardia as a rare complication of interventional closure of ventricular spetal defect and its treatment by radiofrequency catheter ablation: A case report (CARE-compliant)

**DOI:** 10.1097/MD.0000000000019147

**Published:** 2020-03-13

**Authors:** Jing Cao, Qian Xu, Yanbo Liu, Jun Yi, Ruizheng Shi

**Affiliations:** a Department of Cardiovascular Medicine; b Department of Cardiac Surgery, Xiangya Hospital, Central South University, Changsha; c Department of Internal Medicine, Peking Union Medical College Hospital, Beijing, China.

**Keywords:** paroxysmal ventricular tachycardia (PVT), premature ventricular contractions (PVCs), radiofrequency catheter ablation (RFCA), ventricular septal defect (VSD)

## Abstract

**Introduction:**

Trans-catheter closure of peri-membranous ventricular septal defects (VSDs) using Amplatzer-Type devices, has been widely reported in the past decade. We hereby report a rare complication of frequent premature ventricular contractions (PVCs) and paroxysmal ventricular tachycardia (PVT) sustained 48 days after the closure of VSD.More importantly, the arrhythmias were successfully treated with radiofrequency catheter ablation (RFCA) after medical therapy failed to restore and maintain sinus rhythm.

**Patient concerns:**

We reported an 8-year-old boy with frequent PVCs and PVT sustained 48 days after the closure of VSD. The boy has no palpitation, chest distress and other uncomfortable symptoms.

**Diagnosis:**

This patient was diagnosed as frequent PVCs and PVT by Holter monitoring for 24 hours.

**Interventions:**

RFCA was administered.

**Outcomes:**

The patient was discharged 48 hours with no complication and remained asymptomatic 12 months after the ablation.

**Conclusion:**

Radiofrequency ablation helps treat PVCs and PVT in children and has a higher efficacy in restoring and maintaining sinus rhythm.

## 1 Introduction

Trans-catheter closure of peri-membranous ventricular septal defects (VSDs) using Amplatzer-Type devices, has been widely reported in the past decade. It has been proven to have high closure rates, low mortality as well as low rate of complications. Among these rare postoperative complications, complete atrioventricular block is the most common and worrying postoperative complication. Other rare complications include branch block, impulse formation disorder, aortic valve regurgitation and continuous arrhythmia.^[1,2]^ We hereby report a rare complication of frequent premature ventricular contractions (PVCs) and paroxysmal ventricular tachycardia (PVT) sustained 48 days after the closure of VSD in an 8-year-old patient. More importantly, the arrhythmias were successfully treated with radiofrequency catheter ablation (RFCA) after medical therapy failed to restore and maintain sinus rhythm.

## 2 Case

**Figure 1:**
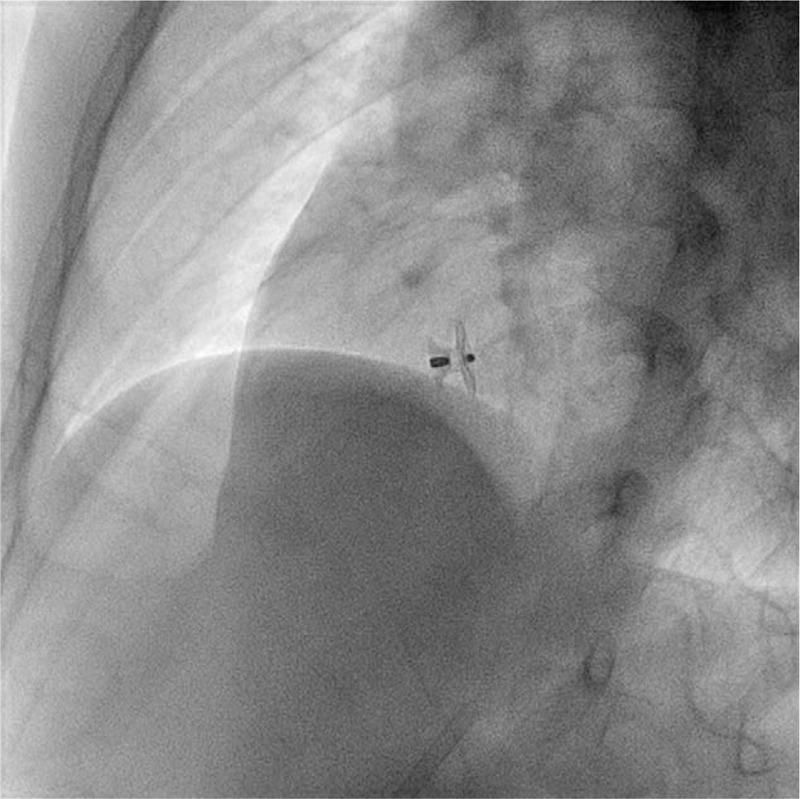
Chest X-ray showed the device location.

An 8-year-old boy was referred to our clinic with PVCs and PVTs observed by 24-hour Holter monitoring. Past history was significant for peri-membranous VSD diagnosed 2 months ago and was closed by an Amplatzer muscular occlude with a waist diameter of 5 mm. On post-op day 1, frequent PVCs were noted on electrocardiogram (ECG). Chest X-ray and echocardiography ruled out device relocation (Fig. 1). Prednisone 5 mg 3 times a day (tid) and Mexiletine 50 mg tid were given to reduce post-op edema and control PVCs. Holter monitoring following the anti-arrhythmic treatment showed PVCs reduced to 33 beats per day. The patient was discharged 6 days post operation and continued to take Mexiletine. PVCs persisted in ECGs in the following 3 weeks. Holter monitoring 48 days post operation revealed a total number of 41,644 PVCs within 24 hours, comprising 31.5% of total heart beats. 3600 PVC couplets, 2367 PVC bigeminy and 2933 episodes of ventricular tachycardia were also noted.

**Figure 2:**
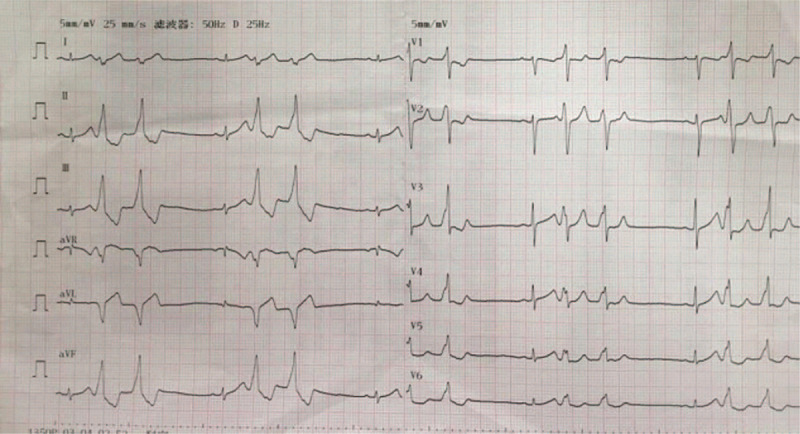
ECG when the patient was admitted to the hospital again after 48 d of the closure of VSD. ECG = electrocardiogram, VSD = ventricular septal defect.

**Figure 3:**
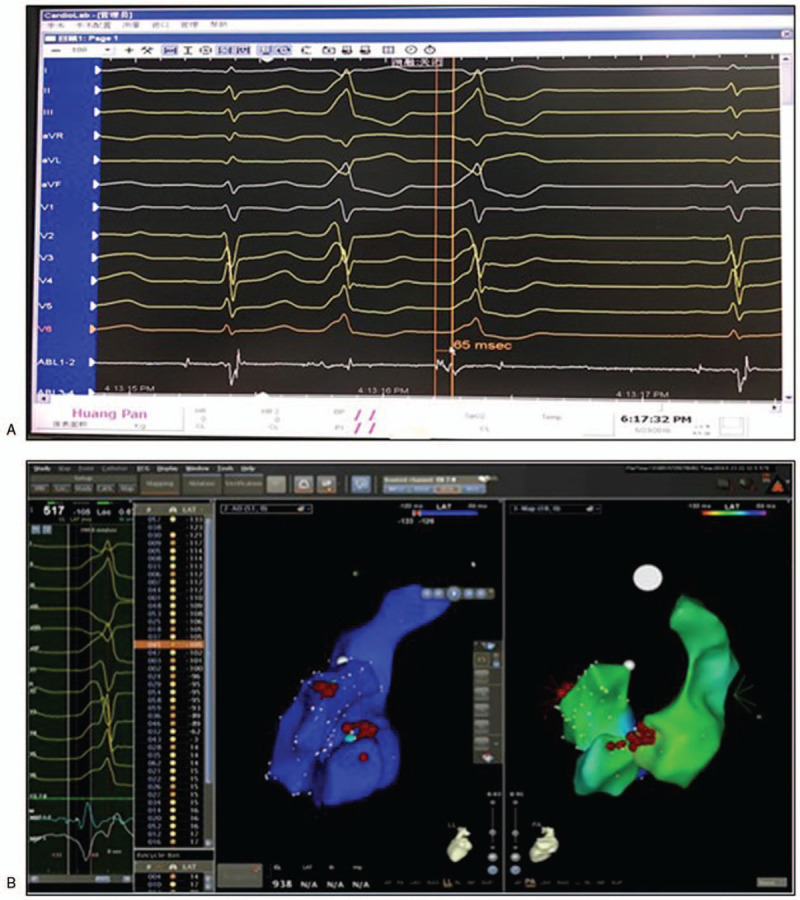
Electroanatomical mapping of patients undergoing ablation.

**Figure 4:**
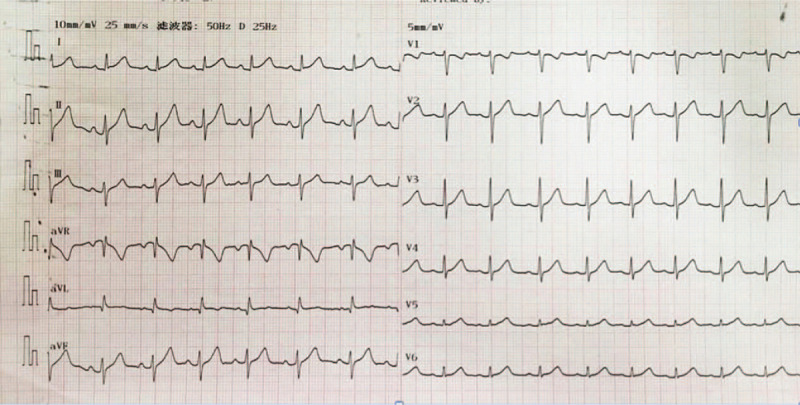
Regular ECGs after the ablation. ECGs = electrocardiograms.

The patient was re-admitted. An ECG was done on admission (Fig. 2). Pre-op labs of complete blood count, cardiac biomarkers, renal and liver function tests all came back negative. Proper inform consent was obtained from the guardians. Mapping and ablation were carried out using a 3.5 mm tip irrigated catheter (ThermoCool SF; Biosense Webster, Diamond Bar, CA) facilitated by an electroanatomic mapping system CARTO 3 (Biosense Webster, Inc). Under the fluoroscopic guidance, XXF catheter was inserted into femoral artery and positioned at aortic sinus. Early activation was spotted in left coronary cusp (LCC) 65 ms pre-QRS with a near perfect pace mapping (97% concordance) (Fig. 3A and B). A safe distance (>10 mm) between the ablation catheter in the LCC and the left main coronary artery was confirmed by coronary angiography. Ablation was then carried out in the LCC with power set to 10 W and maximum temperature to 55°C for 60 seconds around the spot. PVCs disappeared after 6 seconds and were not induced by isoproterenol. The whole procedure lasted for 55 minutes, with fluoroscopic exposure dose of 33 mGy. The patient was discharged 48 hours with no complication and remained asymptomatic 12 months after the ablation. Rregular ECGs (Fig. 4) and Holter monitorings were done and showed sinus rhythm at follow-ups.

## 3 Discussion

This is the first report, to our knowledge, of frequent PVCs and PVTs sustaining 48 days after closure of VSD using Amplatzer-Type devices. Interventional closure of VSD has been performed for over a decade. The most common and worrisome post-operative complication after the interventional closure is complete atrio-ventricular block, which occurs in less than 3% of cases.^[2,3]^ Some suggested it is due to proximity of the conduction system to the margins of the pmVSD, and the compression from the device would cause inflammation leading to electrical disturbance.^[4]^ Other complications, such as device embolization, femoral artery thrombosis as well as transient tachycardia and bradycardia were also been reported.^[4,5]^

The mechanism behind this rare complication remains unclear and needs more research involving complicated modeling. We can only speculate that the early activation foci were stimulated by the maneuver of the catheters or the occluder during the procedure. Another possibility, though less likely, is that the retraction force from the occluder stresses ventricular septum and perhaps the LCC from a distance, causing abnormal electrical excitation. The mechanism maybe a provoking point for further research in electrophysiology.

When the patient had frequent PVCs on the first day after VSD closure, we first treated the patient with drug antiarrhythmia, and Prednisone 5 mg tid. and Mexiletine 50 mg tid were given. However, patients have poor efficacy in drug treatment and PVCs still persist. And when the patient was admitted again, Holter monitoring revealed a total number of more than 10,000 PVCs within 24 hours, and the shape of PVCs was single, which met the surgical indication of RFCA. Therefore, we performed radiofrequency ablation.

In addition, we believe this case further demonstrates the advantages of radiofrequency ablation for PVCs in pediatric patients over the treatment by anti-arrhythmias. Ablation has a higher efficacy in restoring and maintaining sinus rhythm. Kubus et al followed 633 pediatric patients undergoing RFCA for 13.7 years and reported a cumulative success rate of 96.3%.^[6]^ In addition, recurrence of SVT after ablation in pediatric patients was reported to be around 8% with a 34 months follow-up.^[7]^ During the 1-year follow-up in our case, this patient remained asymptomatic and no arrhythmia or any other complication were noticed with follow-up ECGs. Meanwhile, RFCA treatment does not have potential liver/kidney burden or the arrhythmogenic effects associated with antiarrhythmic agents used for adult patient.^[8]^ Lastly, the compliance of medication in pediatric patients has been a major concern which could further decrease the efficacy of antiarrhythmic agents. In our case, the patient need not take any medication on a regular basis during 1-year follow-up.

## 4 Conclusion

In pediatric patients, late onset of frequent PVCs and PVTs is a rare complication after trans-catheter VSD closure. Radiofrequency ablation is efficacious and safe in terminating the arrhythmias and maybe preferred over medical therapy for fewer side effects and better patient compliance.

## Author contributions

**Conceptualization:** Jing Cao, Yanbo Liu.

**Data curation:** Jing Cao, Qian Xu, Yanbo Liu.

**Investigation:** Qian Xu.

**Project administration:** Ruizheng Shi.

**Supervision:** Jun Yi, Ruizheng Shi.

**Validation:** Jun Yi.

**Writing – original draft:** Jing Cao, Qian Xu.

**Writing – review and editing:** Jing Cao, Yanbo Liu.
